# Prognostic Research in Traumatic Brain Injury: Markers, Modeling, and Methodological Principles

**DOI:** 10.1089/neu.2019.6708

**Published:** 2021-08-23

**Authors:** Isabel R.A. Retel Helmrich, Hester F. Lingsma, Alexis F. Turgeon, Jose-Miguel Yamal, Ewout W. Steyerberg

**Affiliations:** ^1^ Department of Public Health, Center for Medical Decision Making, Erasmus MC – University Medical Center Rotterdam, the Netherlands.; ^2^ CHU de Québec – Université Laval Research Centre, Population Health and Optimal Health Practices Research Unit, Trauma – Emergency – Critical Care Medicine, Division of Critical Care Medicine, Faculty of Medicine, Université Laval, Québec City, Québec, Canada.; ^3^ Department of Anesthesiology and Critical Care Medicine, Division of Critical Care Medicine, Faculty of Medicine, Université Laval, Québec City, Québec, Canada.; ^4^ Department of Biostatistics and Data Science, University of Texas School of Public Health, Houston, Texas, USA.; ^5^ Department of Biomedical Data Sciences, Leiden University Medical Center, Leiden, the Netherlands.

**Keywords:** markers, outcome, prognostic research, REMARK, traumatic brain injury, TRIPOD

## Abstract

Prognostic assessment in traumatic brain injury (TBI) is embedded deeply in clinical care. Considering the limitations of current prognostic indicators, there is increasing interest in understanding the role of new biomarkers, and in finding other prognostic indicators of long-term outcomes following TBI. New prognostic indicators may result in the development of more accurate prediction models that could be useful for both risk stratification and clinical decision making. We aimed to review methodological issues and provide tentative guidelines for prognostic research in TBI. Prognostic factor research focuses on the role of a specific patient or disease-related characteristic in relation to outcome. Typically, univariable relations of the prognostic factor are studied, followed by analyses adjusting for other variables related to the outcome. Following existing guidelines, we emphasize the importance of transparent reporting of patient and specimen characteristics, study design, clinical end-points, and statistical analysis. Prognostic model research considers combinations of predictors, with challenges for model specification, estimation, evaluation, validation, and presentation. We highlight modern approaches and opportunities related to missing values, exploration of non-linear effects, and assessing between-study heterogeneity. Prognostic research in TBI can be improved if key methodological principles are adhered to and when research is performed in collaboration among multiple centers to ensure generalizability.

## Introduction

Establishing a reliable prognosis early after traumatic brain injury (TBI) is notoriously difficult because of the heterogeneity of the condition. Clinicians involved in the care of patients with severe TBI are not always in agreement when predicting long-term functional outcomes.^1^ More so, mortality after severe TBI has been observed to be variable across centers in this population, while most TBI patients die following the decision to withdraw life-sustaining therapies.^2^ The lack of appropriate prognostic information was one of the factors shown to influence decisions regarding the level of care for patients with severe TBI.^3^

To predict outcomes after moderate and severe TBI various prediction models have been developed.^4^ Prediction models combine clinical characteristics and data to predict the risk of an outcome for individual patients. Prediction models may support early clinical decision making. They may also facilitate reliable comparison of outcomes among different patient cohorts and variations in results over time. Further, prediction models have been used for risk stratification of patients and covariate adjustment in randomized controlled trials (RCTs).^5,6^

Over the years, several prediction models for moderate and severe TBI were proposed.^4^ Among those, the Corticosteroid Randomisation After Significant Head injury (CRASH) and International Mission for Prognosis and Analysis of Clinical Trials in Traumatic Brain Injury (IMPACT) models formed a contrast to previously developed models.^7,8^ Previous models were commonly developed on relatively small samples, often originated from a single center or region, and lacked external validation.^4^ Simple and more extensive versions of the CRASH and IMPACT prediction models were proposed, with increasing discriminative ability (Supplementary Table S1). Blood biomarkers, imaging biomarkers, and dynamic predictors have been suggested as promising indicators in TBI research, which have the potential to further improve these models.

Although guidelines have been proposed for model development and reporting,^5,9–11^ prognostic research studies in TBI often have methodological limitations.^4,12,13^ We aimed to review methodological issues and provide tentative guidelines for prognostic research in TBI. We first consider prognostic factor research.^11^ Such research focuses on the prognostic role of a single or multiple markers in combination with clinical characteristics and other prognostic indicators. Next, we consider prediction model research.^5^ Because prognostic research is increasingly conducted in collaborative initiatives, such as the International Initiative for Traumatic Brain Injury Research (InTBIR),^14^ we explicitly consider challenges of multi-center data and analyses for model development and validation.

## Methods

Prognostic research studies can be separated into two main categories: studies with a focus on the prognostic role of specific patient- or disease-related characteristics in relation to outcome (“prognostic factor research”), and studies with a focus on the combined effect of various prognostic factors in predicting the outcome (“prediction model research”) (Table 1).^5,11,15^ In prognostic factor studies, we may start with assessing whether the factor is independently associated with the outcome of interest. “Independently” here refers to the association of the prognostic factor with the outcome separate from other prognostic indicators, and typically requires some form of statistical adjustment in the analysis. We may also analyze whether the risk of the outcome uniformly increases or decreases, or has a more complex relationship when considering a continuous predictor. Moreover, we may be interested in a quantification of the incremental predictive value.

**Table 1.** Prognostic Research: Characteristics of Prognostic Factor and Prediction Model Research

Characteristic	Prognostic factor	Prediction model
Research question	1. Is this factor independently associated with the outcome?	How well can we predict outcome based on a combination of prognostic factors?
	2. What is the shape of the association?	
	3. What is the incremental predictive value?	
Effect measure	1. Relative risk (e.g., OR, HR) with 95% confidence interval	Predictive performance, including discrimination (e.g., c statistic) and calibration (e.g., graphical assessment)
	2. Graphical assessment of continuous prognostic factors	
	3. Improvement in performance measures (e.g., c statistic, Nagelkerke *R^2^*)	
Analysis	Univariate analysis and adjusted analysis including other variables related to the outcome	Multivariable modeling and validation

OR, odds ratio; HR, hazard ratio.

In prognostic factor research, it is typical to first study univariable relations of the prognostic factor in a cross-table or regression model, followed by regression analyses adjusting for other variables related to the outcome. The effect measure commonly is relative, for example, an odds ratio (OR) in a logistical regression model, when considering a binary outcome, or hazard ratio (HR) in a Cox regression model, when considering a survival outcome. A *p* value may support claims of statistical significance, which can also be inferred if the 95% confidence interval does not include the value 1. Graphical assessments are helpful to study the shape of an association, while the incremental value can be noted from the improvement in performance measures such as the concordance (c) statistic (equivalent to the area under the receiver operating characteristic [ROC] curve for binary outcomes), or Nagelkerke's *R^2^*.

Whereas prognostic factor research may give rise to speculation on causal effect and mechanism of action, prediction models commonly address a more pragmatic research question: How well can we predict the outcome based on a combination of prognostic factors? In prediction model research, it is typical to analyze combinations of prognostic factors in multivariable models, followed by analyses of predictive performance, including measures for discrimination (e.g., c statistic) and calibration (e.g., graphics and calibration statistics).^6^ The assessment of performance needs validation in independent data. We can distinguish between internal and external validation. With internal validation procedures, such as bootstrap resampling and cross-validation, we aim to correct the performance estimates. In external validation studies, we study the generalizability of the model in different but related settings; for example, by assessing the performance of a proposed model in another cohort.^16,17^

Methodological guidance for prognostic research is dispersed throughout the literature. We take two previously proposed reporting guidelines as a basis: REporting recommendations for tumour MARKer prognostic studies (REMARK), which was originally intended for reporting of marker research in oncology, and Transparent Reporting of a multivariable prediction model for Individual Prognosis Or Diagnosis (TRIPOD), which was proposed for reporting of prediction model development and validation.^9,18^

### Guidance for prognostic factor research

The original REMARK guideline consists of 20 items that need to be reported in studies that focus on one or more prognostic factors (Supplementary Table S2). More specifically, the guideline was developed for prognostic model studies in oncology that include tumor markers. The REMARK guideline is applicable to prognostic factor research in fields other than oncology, and is especially relevant when tissue biomarkers are included as candidate predictors. The guideline was endorsed by multiple journals.^10,18,19^ An “Exploration and Elaboration” document provides more detail on the choice of the items, their relevance, and examples of good practice.^20^ We focus on the items that are most relevant to prognostic factors in TBI research and provide examples for illustration (Table 2).

**Table 2.** Specific Elements for Prognostic Factor Research in TBI, Building on the REMARK Guideline^1^^0^

Topic	Description
Selection of patients	Inclusion and exclusion criteria may vary among studies in relevant aspects, such as age, severity, setting, region, and treatment policies. Inclusion of patients is ideally consecutive.
Prognostic factors considered	Typical prognostic factors may include clinical indicators (vital signs, intracranial pressure, cerebroperfusion pressure); radiological imaging (CT scan, MRI); electrophysiological tests (EEG, SSPEP); and tissue biomarkers (in blood, cerebrospinal fluid). Timing of assessment after trauma, method of acquisition (technology), as well as methods of handling and storage are important to report and address in statistical analyses.
Study design	All candidate variables need to be reported if examined or considered for inclusion in statistical models. Outcome measures must be chosen in relation to the severity of the TBI and may vary from mild to more severe TBI. Rationale for sample size should be provided.
Statistical analysis methods	A core set of predictors needs to be considered for adjustment of prognostic factor associations, depending on the severity of disease and availability of data. Core predictors for moderate and severe TBI include age, motor score (or full GCS), and pupillary reactivity. For mild TBI, the core set is less well defined and may depend on the outcome considered. Missing values in the variables in the core set may be imputed to gain efficiency.
	The relation of the marker to the core set variables needs to be studied; for example, with correlation analyses and graphical inspections.
	Marker values are commonly continuous in nature. The shape of the association with the outcome needs to be examined with sufficiently flexible functions, such as splines, with graphical inspection.
Descriptive results	Consider the flow of patients through each stage of the analysis, with the number of events, and reasons for dropout.
	Describe characteristics in sufficient detail, including demographics, standard prognostic variables, and the prognostic factors considered, including the numbers of missing values.
Statistical results	Univariate and adjusted analyses show the relationship between the marker and outcome, with the estimated association (for example, odds ratio plus confidence interval). Adjustment should be for the core set of variables, irrespective of statistical significance. Incremental predictive value can be indicated by measures for discrimination, such as the increase in c statistic, and overall fit, such as explained variability (*R^2^*).
Interpretation	Results should be interpreted in the context of the pre-specified hypotheses and other relevant studies. Ideally, replication is done in similar studies. Limitations of the study, and implications for future research, need to be considered.

TBI, traumatic brain injury; REMARK, REporting recommendations for tumour *MARKer* prognostic *studies;* CT, computed tomography; MRI, magnetic resonance imaging; EEG, electroencephalography; SSEP, somatosensory evoked potential; GCS, Glasgow Coma Score.

#### Patient selection

When designing prognostic studies, the study population must represent the targeted population for which these models will be used. However, the selection of patients may vary substantially among studies. Inclusion and exclusion criteria may differ among studies in relevant aspects, such as age (pediatric, adult, geriatric), severity (e.g., based on the Glasgow Coma Scale score [GCS]), setting (emergency department, ward, intensive care unit), region (low/middle/high income country), and treatment policies. For example, the CRASH study included 7526 patients from low- and middle-income countries, and 2482 from high-income countries, where mortality at 14 days was 21% versus 16% (*p* < 0.001).^7^ The IMPACT study included 11 cohorts (three RCTs, eight observational studies),^8^ and substantial differences were found in outcome among 265 centers in this study.^21^ In the InTBIR consortium, inclusion criteria also varied substantially among studies, considering the different objectives and targeted populations of TBI patients (See Box 1).

**Box 1. International Initiative for Traumatic Brain Injury Research (InTBIR) Consortium**

InTBIR is a collaborative effort of the European Commission (EC), the National Institutes of Health (NIH), the Canadian Institutes of Health Research (CIHR), the United States Department of Defense (DoD), the Ontario Brain Institute (OBI), and OneMind, which aims to coordinate and leverage international clinical research activities on TBI research. InTBIR's goal is to improve healthcare and lessen the global burden of TBI through the discovery of causal relationships between treatments and clinically meaningful outcomes. InTBIR therefore focuses on collecting, standardizing, and sharing clinical TBI data for comparative effectiveness research.

**Table:** A Selection of InTBIR Studies and their Characteristics

Study	Geographical region(s)	Centers	Inclusion criteria
CREACTIVE	Europe, Israel	72	Moderate to severe TBI
CENTER-TBI	Europe, Israel	59	Clinical diagnosis of TBI; <24 h after injury; clinical indication for CT scan
ADAPT	Australia, Europe, India, New Zealand, South Africa, United States	49	<18 years of age; severe TBI; require ICP monitoring
TRACK-TBI	United States	20	Clinical diagnosis of TBI; clinical indication for CT scan
TBI-Prognosis	Canada	17	Severe TBI; admitted to the intensive care unit
GNRG	Latin America	14	≥13 years of age; Severe TBI; non-penetrating TBI; absence of ICP monitoring
TBIcare	United States	2	≥18 years of age; mild to severe TBI; clinically significant pain over the last 6 months

Information obtained from https://intbir.nih.gov/projects

#### Prognostic factors

Prognostic factors may range from clinical indicators (e.g., disease severity, vital signs, intracranial pressure, cerebral perfusion pressure) to radiological imaging (e.g., computed tomography [CT], magnetic resonance imaging [MRI]), electrophysiological tests (electroencephalogram [EEG], somatosensory evoked potential [SSEP]), and tissue biomarkers (in blood, or cerebrospinal fluid) (Supplementary Table S4). The timing of measurements may vary from the acute phase to several weeks after trauma.

Biomarkers have received increased attention in the last decade. Specifics of the data acquisition need to be considered carefully, and this may be challenging when conducting multi-center studies. Timing and method of acquisition, as well as methods of preservation and storage, are important. Apart from these aspects being reported, they may also need to be addressed in statistical analyses; for example, by adjusting for the time between data acquisition and trauma. If control samples are used, their characteristics also need to be described carefully, including their selection.

The assay methods used should be provided, preferably with a detailed protocol, including specific reagents or kits used, quality control procedures, reproducibility assessments, quantitation methods, and scoring and reporting protocols. Further, it is important to perform assays blinded to the study end-point, for an unbiased assessment.

As an example, biomarkers were sampled at admission up to 24 months post-injury in the Collaborative European NeuroTrauma Effectiveness Research in Traumatic Brain Injury (CENTER-TBI) study. Samples of whole blood, serum, and plasma for genetic, biomarker, and hemostasis analyses were stored in a specific biobank (Pecs, Hungary). A detailed description of the data acquisition, including the timing, method, preservation, and storage has been described elsewhere.^22^

#### Study design

Cohort studies are the preferred design for prognostic research. Ideally, we measure a prognostic factor in a prospective cohort of consecutive patients and evaluate the relationship with the outcome while minimizing potential confounding. Confounding may occur if these prognostic factors (from clinical data or test results) are evaluated according to clinical indications. In TBI research, we might consider a number of different outcomes, depending on the research question and population under study (see Box 2). For efficiency, case-control or nested case-control designs can also be used for prognostic factor studies, especially if measurements are relatively expensive. For reporting, all clinical end-points, and all candidate variables, need to be mentioned if examined or considered for inclusion in any form of statistical analysis. Such a transparent report is essential for proper interpretation of a specific prognostic factor–outcome relationship from a large set of potential relationships as examined in the study. For subjective outcomes measure, blinding of the assessor is important. This means that the assessor should be unaware of the values of the results of the prognostic factors studied.

**Box 2. Selection of Outcomes to Be Considered in Prognosis Research in Traumatic Brain Injury**

**Table:** 

Outcome	Instrument
Mortality	-
Functional status	GOS-E
Generic HRQoL	EQ5-D, SF36, SF12
TBI-specific HRQoL	QoLIBRI, QoLIBRI-OS
Post-concussion symptoms	Rivermead post-concussion questionnaire
Post-traumatic stress disorder	PCL-5
Depression	HADS, PHQ-9
Anxiety	HADS, GAD-7
Neuropsychological testing	GOAT, RAVLT, TMT, CANTAB, 10 m walk and timed up and go
Return to work	-

Adapted from Maas et al., 2014.^22^

GOS-E, Glasgow Outcome Scale-Extended; HRQoL, health-related quality of life; EQ-5D: EuroQol 5 Dimensions Questionnaire; SF12, Short-Form 12; SF36, Short-Form 36; QOLIBRI, Quality of Life after Brain Injury; QOLIBRI-OS, QOLIBRI-Overall Scale; PCL-5, PTSD Check List; HADS, Hospital Anxiety and Depression Scale; PHQ-9, Patient Health Questionnaire; GAD-7, General Anxiety Disorder 7; GOAT, Galveston Orientation and Amnesia Test; RAVLT, Rey Auditory Verbal Learning Test; TMT, Trail Making Test; CANTAB, Cantab neuropsychological assessment tests.

#### Sample size

A methodological and ethical rationale for the sample size should be provided (see Box 3). A formal approach may consider a pre-specified effect size for the prognostic factor, combined with the anticipated distribution of the factor and the end-point. A pragmatic rationale can also be provided.

**Box 3. Example of a Rationale For Sample Size**

**Sample size calculation for the CENTER-TBI study**

The sample size estimate (*n* = 5400) for the CENTER-TBI study was motivated by:

- Practical logistical considerations; higher numbers would imply too large a burden on local, national, and international infrastructure.
- Power calculations for the different strata, targeting comparative effectiveness analyses, assuming a between-center and between-country heterogeneity as identified in previous research (expressed by variance parameter from a random effects model, tau of 0.43).
- Postulated odds ratios for intervention effects of ∼5% improvement in outcome, to be evaluated in comparative effectiveness research.

Overall, a sample size of 5400 subjects would provide statistical power to detect odds ratios of 1.2 associated with differences in process characteristics of specific interventions with a power of 80%.

### Statistical analysis

Some key predictors need to be considered for adjustment of prognostic factor associations (“adjustment model”), with the aim of disentangling the “independent” association of the prognostic factor. The choice of the set will depend on the severity of TBI and the availability of data. Core predictors for moderate and severe TBI may include age, motor score (or full GCS), and pupillary reactivity (based on literature, and the CRASH and IMPACT core models) (Table S1). Other important prognostic factors may include CT characteristics, secondary insults, and biomarker measurements.^23^ For mild TBI, a core set is less well defined and may depend on the outcome considered, such as post-concussive symptoms, neurocognitive functioning, and health-related quality of life. In defining confounders, we should follow epidemiological principles, and not adjust for intermediate factors, which are positioned in between the prognostic factor and the outcome.

The analysis with the adjustment model should ideally be described in detail, not only with respect to the selection of potentially confounding factors, but also on their coding, and the approach to missing values (see Box 4). Further, the relationship of the prognostic factors to the variables in the adjustment model need to be studied, including correlation analyses and graphical inspections. A final issue is how we deal with continuous variables (see Box 5).

**Box 4. Missing Values**

For missing values, multiple imputation has evolved as a standard statistical tool. For many research questions, it is suboptimal to simply drop records with a missing value (complete case analysis). It may be reasonable to drop a variable with high numbers of missing observations.

**Multiple imputation**

- Multiple imputation may often be reasonable for missing values in the variables in the adjustment model, to maximize the available sample size for the adjusted analysis. The assumption is that missingness may be related to other variables, but not to unmeasured confounders. To make this “missing at random” assumption plausible, it is advised to let the imputation model have a rich set of variables: prognostic factors, context factors (e.g. place [site] and time [year] of inclusion), and the outcome.
- Multiple imputation may also be used for the prognostic factor under study. Such imputation may be more controversial, because we may not want to project findings from the prognostic factor: outcome relation in the complete data on the incomplete data. On the other hand, imputation is efficient, especially if the prognostic factor is correlated to other factors. Repeating the imputations multiple times should appropriately capture uncertainty in the process.
- Even more controversial is the imputation of missing outcome data. Statistically, this approach is especially useful if correlates of outcome are available, such that the correlation structure can be exploited. For example, missing 6 month Glasglow Outcome Score (GOS) might be imputed for a patient if 3 and 12 month GOS are available in the data. Again, uncertainty should be captured appropriately by repeating the imputation procedure several times (Richter et al., 2019).^56^ Each imputed data set is analyzed as a complete set, with combination of results according to Rubin's rules (Van Buuren, 2018).^57^

**Box 5. Continuous Variables**

A common approach is to dichotomize prognostic factors as normal/abnormal, or normal/elevated. Such dichotomization implies a loss of information if the original variable was continuous (Royston et al., 2006). This loss can be quantified by comparing model fit with a continuous version of the prognostic factor and a dichotomized version, expressed, for example, as explained variability (*R^2^* statistics). There are many arguments why dichotomization should be avoided in medical research (Royston, 2006).^58^

Instead, the shape of the association of a continuous predictor with the outcome needs to be examined carefully. A linear association may be considered as a starting point. Log transformations are common to consider for biomarkers. Various other types of non-linear functions can be used, which provide greater flexibility, such as square terms, splines, or fractional polynomials. Graphical inspection is also useful to visualize relations that are difficult to grasp from formulas. Differences in fit can be examined, for example, by *R^2^* statistics.

**Continuous variables in the IMPACT study**

**FIG. 1.**
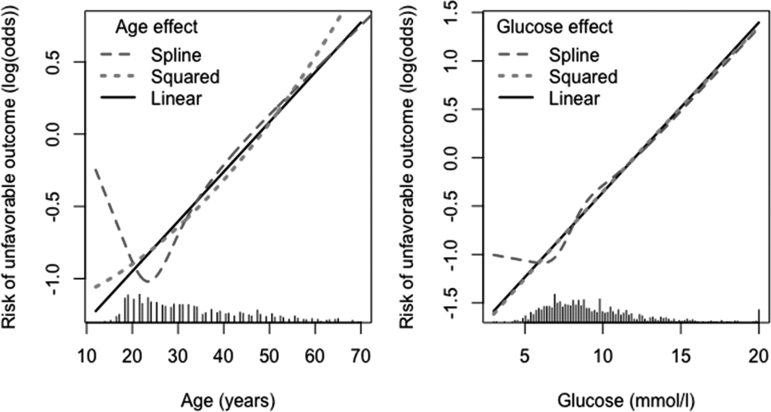
Nonlinearity assessment of continuous variables age (left) and glucose (right) in parts of the International Mission for Prognosis and Analysis of Clinical Trials in Traumatic Brain Injury (IMPACT) study.

Linear relations with outcome were good approximations after assessment of non-linearity using restricted cubic splines for the continuous predictors age and glucose. A positive linear relation was observed for age and glucose, with higher values being associated with poorer prognosis (Fig. 1).

## Results

### Descriptive results

It is important to show the flow of patients through the study, including the number of patients included in each stage of the analysis and the number of dropouts. The number of patients and the number of events need to be clear for each of the analyses performed. Baseline characteristics of the patients must be described in sufficient detail, including distributions of basic demographic characteristics (age, sex, and GCS), standard (disease-specific) prognostic variables, and the prognostic factors considered, including the numbers of missing values.

### Statistical results

Results of univariable and adjusted analyses should be shown to document the relationship between the prognostic factor and the outcome, with the estimated effect size (for example, OR and confidence interval). If imputation of missing observations must be done, modeling results should be compared with the results from a complete case analysis. In a complete case analysis, participants who have missing data on one or multiple predictor variables are excluded. A comparison of the results between imputation and complete case analysis is especially important if participants have missing data on the prognostic factor under study. To evaluate the impact of missing values, it may also be insightful to present patterns of missingness.

Incremental predictive value is often of interest. This can be indicated by measures for discrimination, such as the increase in the concordance statistic (c statistic, or area under the ROC curve [AUC]), and overall fit, such as pseudo *R^2^*.^24^ The c statistic or AUC ranges between 0.50 (no discrimination) and 1.0 (perfect discrimination).

**FIG. 2.**
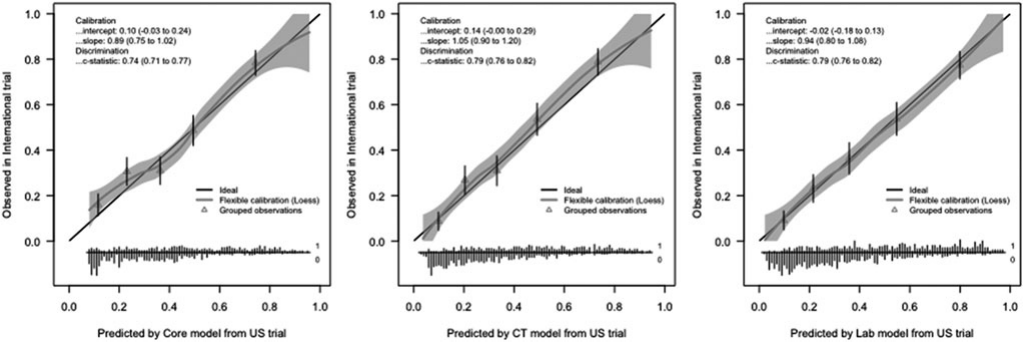
Model performance of the International Mission for Prognosis and Analysis of Clinical Trials in Traumatic Brain Injury (IMPACT) core (left), computed tomography (CT) extended (middle) and laboratory (right) models at external validation in the tirilazad United States trial.

For example, the discriminatory power of the IMPACT models was calculated with a cross-validation procedure within the IMPACT data.^8^ The discriminative ability, indicated with the AUC, increased with increasing model complexity; the AUC was 0.74 for the core, 0.77 for the CT, and 0.79 for the laboratory model for mortality in the tirilazad United States trial (Fig. 2). These results showed the incremental predictive value of the predictors in the CT and laboratory IMPACT models.

### Interpretation

Although studies on prognostic factors or biomarkers often have quite positively framed conclusions, only a few prognostic factors or markers have been validated and proven clinically useful.^25,26^ Results should hence be interpreted with caution, and in the context of the pre-specified hypotheses and other relevant studies. Ideally, replication is done in similar studies. Limitations of the study need to be discussed, as do implications for future research, including the need for validation studies. A prognostic factor may hint at a biological mechanism, opening new avenues for further research, including potential new interventions. A prognostic factor may also simply indicate an association without a deeper explanation. Such a factor may still be useful by allowing for more accurate risk estimation, specifically as a building block with other prognostic factors in prediction models.

### Guidance for prediction model research

The TRIPOD guideline consists of 22 items that need to be reported in a study that focuses on the development and/or validation of a prediction model (Table S3).^9^ Similar to REMARK, the guideline was endorsed by multiple journals,^27,28^ and an extensive “Explanation and Elaboration” document is available.^29^ We focus on the items that are most relevant to prediction models in TBI research (Table 3), and provide examples for illustration.

**Table 3.** Specific Considerations for Prognostic Models in TBI, Building on the TRIPOD Guideline^9^

Topic	Description
Candidate predictors and modeling	All prognostic factors considered in developing or validating the multivariable prediction model need to be mentioned, including how and when they were measured. Discuss approaches to dealing with missing values.
Model development	The modeling approach needs careful description, including dealing with continuous predictors and statistical interactions. Estimation of associations may include shrinkage and penalization techniques to prevent predictions for new patients being too extreme, a problem that occurs with traditional estimation methods. Finally, clustering of data needs to be considered; for example, in a multi-center context, or a multi-cohort context, with fixed or random effect modeling.
Model performance	Important measures include discrimination (c statistic), calibration (graphical assessment, with intercept and slope as key parameters), overall performance (e.g., *R^2^*), and indications of decision quality (decision curve analysis, if clinical decisions are to be supported by the model).
Model validation	Methods for internal validation, such as bootstrap resampling, are important at model development to correct performance estimates for statistical optimism.
	External validation is a stronger test, and starts with a clear description of how the predictions were calculated and a comparison of the validation and development samples. “Internal-external” validation should be considered for multi-center or multi-cohort studies.
Interpretation	Multivariable prediction models have a long tradition in TBI research, but the clinical role of the developed or validated model needs careful discussion; for example, merely informing, benchmarking, or supporting decision making.

TBI, traumatic brain injury; TRIPOD, Transparent Reporting of a multivariable *prediction model* for Individual Prognosis Or Diagnosis.

#### Modeling context

Prediction model development or validation studies share many aspects with prognostic factor research. In both, the selection of patients, the measurements aspects of prognostic factors, and the study design need attention. Prediction models aim to estimate absolute risk and are best developed or validated in cohort studies. The lowest risk of bias is expected in prospective patients and cohorts of consecutive patients. Prospective cohort studies are increasingly being conducted including in the context of the InTBIR initiative.^14^ Data from an RCT has the advantage of careful, standardized, high-quality data collection. However, an RCT typically has specific eligibility criteria, which results in a relatively narrow patient selection. Further, not all consecutive patients may consent to participate. A cohort of patients that is not representative of all consecutive patients is not optimal for prediction modeling. An optimal cohort for prediction models consists of unselected consecutive patients who meet the eligibility criteria for the target population, and is collected through prospective, careful, standardized, and uniformed high-quality data.

The outcome considered for a prediction model should be patient centered. Mortality and the Glasgow Outcome Scale (-Extended) (GOS and GOS-E) 6 months post-injury have often been used, in line with the primary endpoints in RCTs (see Box 2). In prognostic model research, the outcome measure depends on several factors, including the clinical severity of patients, clinical end-point, and the purpose of the model. The GOS-E is a valuable outcome scale in moderate and severe TBI. It is, however, a relatively simplistic scale for assessment of global outcome after TBI, which lacks sensitivity for mild TBI patients. We may, therefore, consider including other outcomes, such as health-related quality of life, post-concussive symptoms, and neurocognitive functioning, especially for patients following mild TBI.

As for prognostic factor research, the sample size needs to be justified. In addition to reasoning based on the anticipated effect size of a prognostic factor, sample size can also be motivated by rules of thumb, such as having at least 10 events per candidate predictor.^6^

#### Candidate predictors

The CRASH and IMPACT models considered only three to four predictors for their Basic and Core models, respectively (Table S1). This narrow selection of predictors was motivated by a literature review to identify the most common and strong key predictors in moderate to severe TBI.^13^ A wider selection may be of interest to improve the predictive ability. It may then make sense to consider extensions on the small set of key predictors rather than starting a “*de novo* model” building exercise. In mild TBI, prediction models and key predictors are less well established, which may motivate a more exploratory approach.

If the number of candidate predictors is limited (e.g., <20), this is considered to be modeling of low-dimensional data. A different and challenging field is that of high- dimensional data, where numbers of candidate predictors may exceed the numbers of patients (“p>n situation,” see Box 6). In any case, all candidate predictors that are used in developing or validating the multivariable prediction model should be listed, with a description of how and when they were measured.

**Box 6. Modeling Techniques**

Prognostic models are usually developed with multivariable regression techniques on data from (prospective) cohort studies. Prognostic models for traumatic brain injury (TBI) most commonly predict binary outcomes (e.g., mortality, unfavorable outcome). For such binary outcomes, the logistical regression model is the most widely used statistical technique. Recently, modern modeling techniques, such as penalized estimation and machine learning, are gaining increased attention. Machine learning techniques aim to learn more directly from the data, without assuming some type of underlying statistical model. These techniques are more flexible than traditional modeling techniques (e.g., they can adjust the complexity of the method according to the data, and can capture complex relationships between the factors and the outcome). However, more data is required to obtain accurate estimates of model parameters, and reduce the risk of overfitting.

**Table:** Traditional and Modern Modeling Techniques

Modeling techniques	Methods
Regression	Logistical regression
	Generalized additive model
Penalized estimation	Ridge regression
	LASSO
	Elastic net
Machine learning techniques	Neural network
	Random forest
	Support vector machine

As was discussed for prognostic factors, missing values are a key issue. A candidate predictor may be excluded from further modeling if it has many missing values (e.g., >50% missing values). Traditional multivariable statistical analysis may suffer severely from missing values: a single missing value in one of the candidate predictors causes the full patient record to be discarded. Multiple imputation is a well-known strategy to fill in missing values. It allows for statistically appropriate use of records with one or more missing values, and should hence be preferred over a complete case analysis.

#### Model development

Prognostic models are typically developed using regression analysis. Recently, machine learning approaches, such as support vector machines, random forests, and neural nets, have gained increased attention. It is yet unclear whether and in which scenarios such methods outperform regression analysis in prediction of outcome following TBI.^30^

Modeling a combination of predictors poses challenges that include potential non-linear associations, interaction effects of predictors, optimal estimation, and dealing with clustering. When we consider a continuous predictor as a linear term in a prediction model, we assume that the effect is the same at each part of the range of the predictor. For example, we may assume that the effect of being 10 years older is the same at the age of 40 (50 vs. 40) and 70 (80 vs. 70) years for patients following TBI. If a non-linear relation is expected, we can use flexible functions such as splines or fractional polynomials in regression models. Modern modeling and algorithmic approaches, such as generalized additive models (GAM) or support vector machines (SVMs), may include even more flexible high-dimensional smoothness in the relations of continuous predictors to the outcome.

Combinations of prognostic factors may have differential effects, meaning that statistical interactions may be present. Multiplicity is a threat in attempts of modeling such interactions. For example, five predictors imply that 10 potential two-way interactions could be studied, while ignoring higher-order interactions. The number of potential interactions rises quickly with a large number of predictors (e.g., 45 for 10 predictors, and 190 for 20 predictors). Currently, available prediction models often ignore such potential interactions and merely rely on the main effects of predictors. If assessed, it may be useful to perform overall tests of significance (e.g., considering all potential interactions with age and sex).^31^ If such a single overall test does not show significant results, we may decide to ignore the interactions. Alternatively, tree-based methods such as like Classification And Regression Trees (CART) and random forests indirectly consider interactions between factors. However, these methods are at risk for overfitting.^30^

Another challenge lies in the optimal estimation of prognostic associations. Statistical overfitting of available data is a key problem in prediction models: patterns in the data are described that do not generalize outside the specific data set considered.^32^ As the complexity of the model (e.g., the number of coefficients or parameters estimated) increases, there is a greater risk for overfitting. Such overfitting may be reduced by reducing the number of examined prognostic associations in a model. Regression coefficients can be shrunk toward zero for less extreme and more stable predictions.^33,34^ Similarly, penalization procedures can be followed, such as ridge regression, and penalized estimation. A particularly promising approach is the Least Absolute Shrinkage and Selection Operator (LASSO), which shrinks some coefficients to zero, hence effectively reducing the set of candidate predictors. Such selection through penalization is a statistical improvement over classical stepwise selection methods (e.g., backward stepwise selection based on *p* values). Stepwise selection methods have many disadvantages; for example, these methods lead to too extreme estimates of the effect of selected predictors.^6^

Finally, the statistical analysis may need to consider the clustering of data; for example, in the context of a multi-center or multi-cohort study. Stratification by cluster can be achieved by a fixed effect approach (by conditioning on the cluster; e.g., with dummy variables for the studies), or by random effect modeling (also known as hierarchical modeling, or mixed effect modeling). Random effect modeling is advised in instances of a larger number of clusters; for example, more than five clusters, which allows for a quantification of the between-cluster heterogeneity. Heterogeneity is commonly found in the baseline risk, whereas heterogeneity in prognostic effects may be less relevant; specifically, if clusters are similar in basic attributes (setting, inclusion criteria).^35^ Updating of a model to a specific setting may be motivated by substantial heterogeneity across settings.^36,37^

#### Model performance

Performance of prediction models is most commonly assessed with respect to discriminative ability as in the question of how well we can separate low risk (favorable outcome) from high-risk patients (unfavorable outcome). Discriminative ability is then measured with a concordance (c) statistic (equivalent to the AUC for binary outcomes). The c statistic is a rank order statistic that ranges between 0.5 (no discrimination) and 1 (perfect discrimination). Limits for “satisfactory” or “good” discrimination are inherently subjective, and context dependent. Values at ∼0.8 have been achieved for rather simple prediction models (Basic CRASH and Core IMPACT models, Table S1). We note that the discriminative ability depends not only on model characteristics (strong prognostic associations), but also on the heterogeneity of the sample of patients (between patient- differences and case mix). Hence, discrimination was substantially higher in unselected cohort studies than in RCTs.^38^

Performance can further be quantified by calibration, as by asking if close to *x* of 100 patients with a risk prediction of *x*% have the outcome. A graphical assessment of calibration is attractive, with predictions on the x-axis and the outcome on the y-axis. Perfect predictions should be on the 45 degree line. For binary outcomes, the plot contains 0 and 1 values for the y-axis. Smoothing techniques can be used to estimate the observed probabilities of the outcome in relation to the predicted probabilities (e.g., using the locally estimated scatterplot smoothing [LOESS] algorithm or polynomials).^39,40^

The calibration plot can be characterized by an intercept *a*, which indicates whether predictions are systematically too low or too high (“calibration-in-the-large”), and a calibration slope *b*, which should be 1.^41^ At model development, a = 0 and b = 1 for classical regression models. At validation, calibration-in-the-large problems are common, as well as *b* < 1, reflecting overfitting of a model.

Performance of the CRASH model was assessed in terms of calibration (calibration graph) and discrimination (c statistic). In the derivation sample, the CRASH models showed excellent discrimination, with c statistics >0.80. Moreover, the models showed good calibration graphically.

Overall performance measures relate to goodness of fit. For binary outcomes, a model's goodness of fit can be assessed through measures similar to overall performance measures for linear models that indicate the explained variability, here labeled “pseudo *R^2^*.” Pseudo *R^2^* is based on the improvement in model likelihood over a null model.^42^ These measures are especially useful for assessing incremental value of predictors. Several variants are available, including Nagelkerke's and McFadden's *R^2^*, which both provide for a natural scaling between 0 and 100%.^43^

Recent developments include decision-analytic approaches that explicitly consider the relative weight of false-positive and true-positive classifications. Such weighting is essential if prediction models are used to help guide clinical decisions. However, prediction models have not been perceived clinically useful to help guide decisions with regard to level of care. However, they could be eventually used as part of decision support tools or aid for level of care decisions. “Decision curve analysis” has been proposed as a technique to quantify clinical utility, if clinical decisions are to be supported by the model. Here, the clinical utility of a prediction model, or the extension of a model with a prognostic factor, is examined over a range of plausible decision thresholds.^44^

#### Model validation

Model performance can be estimated from the sample where the model was developed (“apparent performance”). Such assessments are usually optimistic because the model was optimized for the same data where performance was evaluated. Methods for internal validation, such as bootstrap resampling or cross-validation, are important during model development to correct the performance estimates.^45^ Bootstrap procedures can estimate statistical optimism if all modeling steps are replayed per bootstrap resample, such as selection from a set of candidate predictors.^46^ Both bootstrap resampling and cross-validation use all available data for model development and are therefore preferred methods for internal validation. A split sample approach uses only part of the data for model development and is not recommended, because of inefficiency.^45^

External validation is a stronger test for a prediction model. Such an analysis starts with a clear description of how the predictions were calculated and a comparison of the validation and development samples. Specifically, the heterogeneity in case mix is relevant: more heterogeneous validation samples will increase the expected discrimination.^47^ Temporal validation includes validation in a more recent data set, whereas geographic validation includes validation in another place. Large scale multi-center studies provide good opportunities for what has been labeled “internal-external validation.” With internal-external validation, every center or cohort is left out once when developing the models. Models are evaluated in the centers or cohorts that were not used for model development. This process is repeated until all participants have been used for model validation, so that all centers are left out once, and model performance is estimated over all validations. This procedure is a variant of cross-validation, a technique that can also be used for internal validation. At internal validation, parts of the data are left out at random, whereas internal-external validation leaves non-random parts of the data out. A sufficient number of events is required for reliable assessment of performance.^48^

As an example, the IMPACT models were cross-validated across IMPACT cohorts and externally validated in the CRASH trial.^6^ Across the IMPACT cohorts, the best performance was seen for the three observational studies, with AUCs >0.80, whereas the RCTs showed lower discriminative abilities. At external validation in the CRASH trial, the discriminatory ability of the models increased with increasing complexity; the AUCs ranged between 0.77 and 0.80.

#### Interpretation

The clinical role of the model needs careful consideration. In the design of RCTs, prediction models may assist in patient selection or stratification.^49^ In the analysis of RCTs, prognostic factors can be used for covariate adjustment. In that case, we advise that the model be refitted in the sample under study, preferably with the prognostic factors as individual variables.^50^ One of the aims of this adjustment is to correct for any imbalance that may have arisen (by chance) between the treatment arms. Moreover, the statistical power of an adjusted analysis of the treatment effect may be larger than that of an unadjusted analysis.^51^

In observational studies, a prediction model may be used for confounder adjustment in comparing outcomes among centers or at a patient level. Prediction models may also serve as a reference in the evaluation of the incremental value of a new prognostic factor. A prognostic factor should be added to the reference model for a fair evaluation.^52^ Such an evaluation should also consider the time of assessment. For example, when prognostic factors are measured during the first 3 days of hospital admission, a reference model can be based on all available information until 3 days rather than focusing on the admission phase.

Validity of estimated associations and baseline risk is required if predictions are used to provide prognostic estimates to patients or relatives, benchmarking, or decision support. Similarly, validity of predictions is essential if models are used to support the decision-making process at the bedside. For example, models are used to decide whether or not a patient should have a CT performed following mild TBI when consulting in the emergency room.^53^

Model validations in various medical fields have shown that baseline risk often varies among settings.^54^ For TBI, substantial between-center differences in baseline risk have been shown.^55^ Using a prediction model in a specific setting hence requires consideration of the plausibility of applying absolute risk predictions from the development setting. Ideally, a validation study is performed, to verify whether the average observed and predicted risks are similar (calibration-in-the-large, reflecting correctness of baseline risk). Often, we find that some statistical updating of the baseline risk is needed.^37^

### Summary

Establishing a reliable prognosis early after TBI is challenging because of several factors, including the heterogeneity of the condition. Further, prognostic research in TBI often has methodological limitations, which has resulted in a lack of reliable prognostic information for patients with moderate and severe TBI. We aimed to review methodological issues and provide tentative guidelines for prognostic research in TBI. For this purpose, we have considered two existing reporting guidelines: the REMARK and TRIPOD guidelines, from which advice on appropriate methods can be inferred. For prognostic factor research, we emphasize the importance of transparent reporting of patient and specimen characteristics, study design, clinical end-points, and statistical analysis. Prediction model research especially brings challenges for model specification, estimation, evaluation, validation, and presentation. The TRIPOD guidelines underscore the importance of transparent reporting of these aspects of model development and validation. Further, we have highlighted modern approaches and opportunities related to missing values, exploration of non-linear effects, and assessing between-study heterogeneity by leave-one-study-out cross-validation.

## Discussion

Our review presents various methodological aspects of prognostic research and may provide a solid foundation for future studies of prognostic factors and prediction models in TBI. For prognostic factor research, we took the REMARK guidelines as a foundation. These guidelines have been developed by methodological experts and have been received positively by other scientists and editors of journals.^10,18,19^ Similarly, we used TRIPOD as a foundation for methodological guidance for prediction model research.^9,27,28^ These guidelines are primarily intended for transparent reporting. Additionally, advice on appropriate methods can be inferred from the items listed, and from the motivation for the inclusion of the items in these guidelines. Also, more in-depth material is available in the “Explanation and Elaboration” documents of these guidelines.^20,29^

In prognostic research it is important to carefully describe the selection of patients, the prognostic factors considered, and the study design. Previous reviews showed poor methodological quality of many model development studies in TBI,^12,13^ and improvements can be made with respect to dealing with missing values (multiple imputation), assessment of non-linear relations (using splines or other flexible functions), and estimation of prognostic associations (e.g., using LASSO). For prediction models, modern machine learning algorithms may prove to be useful for modeling of high-dimensional data. No benefit is expected from such methods in low-dimensional data.^30^ Classical regression models may then be adequate, especially if the selection from candidate predictors is done carefully, and modern shrinkage or penalization techniques are used to prevent too extreme and too optimistic predictions.

Validation of prognostic claims is essential in prognostic factor research and prediction model research. Prediction models need external validation to assess discriminative ability and reliability (calibration) of predictions in new settings. Heterogeneous model performance can be interpreted as a warning signal that simple universal applicability of a “global” prediction model should be reconsidered, and this indicates that the prediction model should be updated per setting.^55^ Various methods are available to update models to a specific setting, including calibration-in-the large, adjustment of all regression coefficients, updating of individual predictor effects, and extending the model with new predictors.^37^ Recent collaborative efforts, such as the InTBIR consortium, provide opportunities for validation. For prognostic factors, we suggest providing forest plots with estimates per study, as is standard for genomic analyses. For prediction models, we suggest internal-external validation procedures, in which performance is assessed in a study that is left out of the model development process.

Prognostic research in TBI can be improved if the described key methodological principles are adhered to, and when research is performed in collaboration among multiple centers. Recent collaborative initiatives provide new opportunities for large-scale studies with cross-validation of promising findings.

## Supplementary Material

Supplemental data

Supplemental data

Supplemental data

Supplemental data

## References

[1] Turgeon, A.F., Lauzier, F., Burns, K.E.A., Meade, M.O., Scales, D.C., Zarychanski, R., Moore, L., Zygun, D.A., McIntyre, L.A., and Kanji, S. (2013). Determination of neurologic prognosis and clinical decision making in adult patients with severe traumatic brain injury: a survey of Canadian intensivists, neurosurgeons, and neurologists. Crit. Care Med. 4, 1086–1093 10.1097/CCM.0b013e318275d046 23385104

[2] Turgeon, A.F., Lauzier, F., Simard, J.-F., Scales, D.C., Burns, K.E.A., Moore, L., Zygun, D.A., Bernard, F., Meade, M.O., and Dung, T.C. (2011). Mortality associated with withdrawal of life-sustaining therapy for patients with severe traumatic brain injury: a Canadian multicentre cohort study. CMAJ 14, 1581–1588 10.1503/cmaj.101786 PMC3185074 21876014

[3] Turgeon, A.F., Dorrance, K., Archambault, P., Lauzier, F., Lamontagne, F., Zarychanski, R., Fowler, R., Moore, L., Lacroix, J., and English, S. (2019). Factors influencing decisions by critical care physicians to withdraw life-sustaining treatments in critically ill adult patients with severe traumatic brain injury. CMAJ 24, E652–E663 10.1503/cmaj.190154 PMC6581526 31209132

[4] Dijkland, S.A., Foks, K.A., Polinder, S., Dippel, D.W.J., Maas, A., Lingsma, H., and Steyerberg, E.W. (2020). Prognosis in moderate and severe traumatic brain injury: a systematic review of contemporary models and validation studies. J. Neurotrauma 37, 1–13. [Epub ahead of print.] 31099301 10.1089/neu.2019.6401

[5] Steyerberg, E.W., Moons, K.G.M., van der Windt, D.A., Hayden, J.A., Perel, P., Schroter, S., Riley, R.D., Hemingway, H., Altman, D.G., and Group, P. (2013). Prognosis Research Strategy (PROGRESS) 3: prognostic model research. PLoS Med 2, e1001381 10.1371/journal.pmed.1001381 PMC3564751 23393430

[6] Steyerberg, E.W. (2008). Clinical Prediction Models: A Practical Approach to Development, Validation, and Updating. Springer Science & Business Media: New York

[7] MRC CRASH Trial Collaborators, Perel, P., Arango, M., Clayton, T., Edwards, P., Komolafe, E., Poccock, S., Roberts, I., Shakur, H., Steyerberg, E., and Yutthakasemsunt, S. (2008). Predicting outcome after traumatic brain injury: practical prognostic models based on large cohort of international patients. BMJ 7641, 425–429 10.1136/bmj.39461.643438.25 PMC2249681 18270239

[8] Steyerberg, E.W., Mushkudiani, N., Perel, P., Butcher, I., Lu, J., McHugh, G.S., Murray, G.D., Marmarou, A., Roberts, I., and Habbema, J.D.F. (2008). Predicting outcome after traumatic brain injury: development and international validation of prognostic scores based on admission characteristics. PLoS Med 8, e165 10.1371/journal.pmed.0050165 PMC2494563 18684008

[9] Collins, G.S., Reitsma, J.B., Altman, D.G., and Moons, K.G.M. (2015). Transparent reporting of a multivariable prediction model for individual prognosis or diagnosis (TRIPOD): the TRIPOD statement. BMC Med. 1, 1 10.1186/s12916-014-0241-z PMC4284921 25563062

[10] McShane, L.M., Altman, D.G., Sauerbrei, W., Taube, S.E., Gion, M., and Clark, G.M. (2005). Reporting recommendations for tumor marker prognostic studies (REMARK). J. Natl. Cancer Inst. 16, 1180–1184 10.1093/jnci/dji237 16106022

[11] Riley, R.D., Hayden, J.A., Steyerberg, E.W., Moons, K.G.M., Abrams, K., Kyzas, P.A., Malats, N., Briggs, A., Schroter, S., and Altman, D.G. (2013). Prognosis Research Strategy (PROGRESS) 2: prognostic factor research. PLoS Med 2, e1001380 10.1371/journal.pmed.1001380 PMC3564757 23393429

[12] Perel, P., Edwards, P., Wentz, R., and Roberts, I. (2006). Systematic review of prognostic models in traumatic brain injury. BMC Med. Inform. Decis. Mak. 1, 38 10.1186/1472-6947-6-38 PMC1657003 17105661

[13] Mushkudiani, N.A., Hukkelhoven, C.W.P.M., Hernández, A.V., Murray, G.D., Choi, S.C., Maas, A.I.R., and Steyerberg, E.W. (2008). A systematic review finds methodological improvements necessary for prognostic models in determining traumatic brain injury outcomes. J. Clin. Epidemiol. 4, 331–343 10.1016/j.jclinepi.2007.06.011 18313557

[14] Tosetti, P., Hicks, R.R., Theriault, E., Phillips, A., Koroshetz, W., Draghia, A., and Workshop Participants (2013). Toward an international initiative for traumatic brain injury research. J. Neurotrauma 14, 1211–1222 10.1089/neu.2013.2896 PMC3713440 23731282

[15] Bouwmeester, W., Zuithoff, N.P.A., Mallett, S., Geerlings, M.I., Vergouwe, Y., Steyerberg, E.W., Altman, D.G., and Moons, K.G.M. (2012). Reporting and methods in clinical prediction research: a systematic review. PLoS Med. 5, e1001221 10.1371/journal.pmed.1001221 PMC3358324 22629234

[16] Justice, A.C., Covinsky, K.E., and Berlin, J.A. (1999). Assessing the generalizability of prognostic information. Ann. Intern. Med. 6, 515–524 10.7326/0003-4819-130-6-199903160-00016 10075620

[17] Steyerberg, E.W. and Harrell, F.E. (2016). Prediction models need appropriate internal, internal–external, and external validation. J. Clin. Epidemiol. 69, 245–247 25981519 10.1016/j.jclinepi.2015.04.005 PMC5578404

[18] Hayes, D.F., Ethier, S., and Lippman, M.E. (2006). New guidelines for reporting of tumor marker studies in breast cancer research and treatment: REMARK. Breast Cancer Res. Treat. 2, 237–238 10.1007/s10549-006-9253-5 16773436

[19] McShane, L.M., Altman, D.G., Sauerbrei, W., Taube, S.E., Gion, M., and Clark, G.M. (2006). REporting recommendations for tumor MARKer prognostic studies (REMARK). Breast Cancer Res. Treat. 2, 229–235 10.1007/s10549-006-9242-8 16932852

[20] Altman, D.G., McShane, L.M., Sauerbrei, W., and Taube, S.E. (2012). Reporting recommendations for tumor marker prognostic studies (REMARK): explanation and elaboration. BMC Med. 1, 51 10.1186/1741-7015-10-51 PMC3362748 22642691

[21] Lingsma, H.F., Roozenbeek, B., Li, B., Lu, J., Weir, J., Butcher, I., Marmarou, A., Murray, G.D., Maas, A.I.R., and Steyerberg, E.W. (2011). Large between-center differences in outcome after moderate and severe traumatic brain injury in the international mission on prognosis and clinical trial design in traumatic brain injury (IMPACT) study. J. Neurosurg. 3, 601–608 10.1227/NEU.0b013e318209333b 21311293

[22] Maas, A.I.R., Menon, D.K., Steyerberg, E.W., Citerio, G., Lecky, F., Manley, G.T., Hill, S., Legrand, V., and Sorgner, A. (2014). Collaborative European NeuroTrauma Effectiveness Research in Traumatic Brain Injury (CENTER-TBI) a prospective longitudinal observational study. J. Neurosurg. 1, 67–80 10.1227/NEU.0000000000000575 25525693

[23] Winn, H.R. (2011). Youmans Neurological Surgery E-Book, Elsevier Health Sciences: Philadelphia, PA

[24] Steyerberg, E.W., Pencina, M.J., Lingsma, H.F., Kattan, M.W., Vickers, A.J., and Van Calster, B. (2012). Assessing the incremental value of diagnostic and prognostic markers: a review and illustration. Eur. J. Clin. Invest. 2, 216–228 10.1111/j.1365-2362.2011.02562.x PMC3587963 21726217

[25] Ioannidis, J.P.A. (2013). Biomarker failures. Clin. Chem. 1, 202–204 10.1373/clinchem.2012.185801 22997282

[26] Ioannidis, J.P.A. and Bossuyt, P.M.M. (2017). Waste, leaks, and failures in the biomarker pipeline. Clin. Chem. 5, 963–972 10.1373/clinchem.2016.254649 28270433

[27] Localio, A.R. and Stack, C.B. (2015). TRIPOD: a new reporting baseline for developing and interpreting prediction models. Ann. Intern. Med. 1, 73–74 10.7326/M14-2423 25560717

[28] Moons, K.G.M., Altman, D.G., Reitsma, J.B., and Collins, G.S. (2015). New guideline for the reporting of studies developing, validating, or updating a multivariable clinical prediction model: the TRIPOD statement. Adv. Anat. Pathol. 5, 303–305 10.1097/PAP.0000000000000072 26262512

[29] Moons, K.G.M., Altman, D.G., Reitsma, J.B., Ioannidis, J.P.A., Macaskill, P., Steyerberg, E.W., Vickers, A.J., Ransohoff, D.F., and Collins, G.S. (2015). Transparent Reporting of a multivariable prediction model for Individual Prognosis or Diagnosis (TRIPOD): explanation and elaboration. Ann. Intern. Med. 1, W1–W73 10.7326/M14-0698 25560730

[30] van der Ploeg, T., Nieboer, D., and Steyerberg, E.W. (2016). Modern modeling techniques had limited external validity in predicting mortality from traumatic brain injury. J Clin Epidemiol. 78, 83–89 26987507 10.1016/j.jclinepi.2016.03.002

[31] Harrell Jr, F.E. (2015). Regression Modeling Strategies: With Applications to Linear Models, Logistic and Ordinal Regression, and Survival Analysis. Springer: New York, NY

[32] Babyak, M.A. (2004). What you see may not be what you get: a brief, nontechnical introduction to overfitting in regression-type models. Psychosom. Med. 3, 411–421 10.1097/01.psy.0000127692.23278.a9 15184705

[33] Copas, J.B. (1983). Regression, prediction and shrinkage. J R STAT SOC A STAT: Series B (Methodological), 45, 311–335

[34] Van Houwelingen, J.C. (2001). Shrinkage and penalized likelihood as methods to improve predictive accuracy. Stat. Neerl. 1, 17–34

[35] Austin, P.C., van Klaveren, D., Vergouwe, Y., Nieboer, D., Lee, D.S., and Steyerberg, E.W. (2017). Validation of prediction models: examining temporal and geographic stability of baseline risk and estimated covariate effects. Diagn. Progn. Res. 1, 12 29350215 10.1186/s41512-017-0012-3 PMC5770216

[36] Steyerberg, E.W., Borsboom, G.J.J.M., van Houwelingen, H.C., Eijkemans, M.J.C., and Habbema, J.D.F. (2004). Validation and updating of predictive logistic regression models: a study on sample size and shrinkage. Stat. Med. 16, 2567–2586 10.1002/sim.1844 15287085

[37] Vergouwe, Y., Nieboer, D., Oostenbrink, R., Debray, T.P.A., Murray, G.D., Kattan, M.W., Koffijberg, H., Moons, K.G.M., and Steyerberg, E.W. (2017). A closed testing procedure to select an appropriate method for updating prediction models. Stat. Med. 28, 4529–4539 10.1002/sim.7179 27891652

[38] Roozenbeek, B., Lingsma, H.F., Lecky, F.E., Lu, J., Weir, J., Butcher, I., McHugh, G.S., Murray, G.D., Perel, P., and Maas, A.I.R. (2012). Prediction of outcome after moderate and severe traumatic brain injury: external validation of the IMPACT and CRASH prognostic models. Crit. Care Med. 5, 1609 10.1097/CCM.0b013e31824519ce PMC3335746 22511138

[39] Austin, P.C., and Steyerberg, E.W. (2014). Graphical assessment of internal and external calibration of logistic regression models by using loess smoothers. Stat. Med. 3, 517–535 10.1002/sim.5941 PMC4793659 24002997

[40] Nattino, G., Finazzi, S., and Bertolini, G. (2014). A new calibration test and a reappraisal of the calibration belt for the assessment of prediction models based on dichotomous outcomes. Stat. Med. 14, 2390–2407 10.1002/sim.6100 24497413

[41] Cox, D.R. (1958). Two further applications of a model for binary regression. Biometrika 3/4, 562–565

[42] Hemmert, G.A.J., Schons, L.M., Wieseke, J., and Schimmelpfennig, H. (2018). Log-likelihood-based pseudo-R 2 in logistic regression: deriving sample-sensitive benchmarks. Sociol. Methods Res. 3, 507–531

[43] Smith, T.J., and McKenna, C.M. (2013). A comparison of logistic regression pseudo R2 indices. Multiple Linear Regression Viewpoints 2, 17–26

[44] Vickers, A.J., and Elkin, E.B. (2006). Decision curve analysis: a novel method for evaluating prediction models. Med. Decis. Making 6, 565–574 10.1177/0272989X06295361 PMC2577036 17099194

[45] Steyerberg, E.W., Harrell Jr, F.E., Borsboom, G.J.J.M., Eijkemans, M.J.C., Vergouwe, Y., and Habbema, J.D.F. (2001). Internal validation of predictive models: efficiency of some procedures for logistic regression analysis. J. Clin. Epidemiol. 8, 774–781 10.1016/s0895-4356(01)00341-9 11470385

[46] Steyerberg, E.W., Bleeker, S.E., Moll, H.A., Grobbee, D.E., and Moons, K.G.M. (2003). Internal and external validation of predictive models: a simulation study of bias and precision in small samples. J. Clin. Epidemiol. 5, 441–447 10.1016/s0895-4356(03)00047-7 12812818

[47] Debray, T.P.A., Vergouwe, Y., Koffijberg, H., Nieboer, D., Steyerberg, E.W., and Moons, K.G.M. (2015). A new framework to enhance the interpretation of external validation studies of clinical prediction models. J. Clin. Epidemiol. 3, 279–289 10.1016/j.jclinepi.2014.06.018 25179855

[48] Vergouwe, Y., Steyerberg, E.W., Eijkemans, M.J.C., and Habbema, J.D.F. (2005). Substantial effective sample sizes were required for external validation studies of predictive logistic regression models. J. Clin. Epidemiol. 5, 475–483 10.1016/j.jclinepi.2004.06.017 15845334

[49] Roozenbeek, B., Maas, A.I.R., Lingsma, H.F., Butcher, I., Lu, J., Marmarou, A., McHugh, G.S., Weir, J., Murray, G.D., and Steyerberg, E.W. (2009). Baseline characteristics and statistical power in randomized controlled trials: selection, prognostic targeting, or covariate adjustment? Crit. Care Med. 10, 2683–2690 10.1097/ccm.0b013e3181ab85ec 19885979

[50] Turner, E.L., Perel, P., Clayton, T., Edwards, P., Hernández, A.V., Roberts, I., Shakur, H., Steyerberg, E.W., and CRASH Trial Collaborators (2012). Covariate adjustment increased power in randomized controlled trials: an example in traumatic brain injury. J. Clin. Epidemiol. 5, 474–481 10.1016/j.jclinepi.2011.08.012 PMC3589911 22169080

[51] Hernández, A.V., Steyerberg, E.W., and Habbema, J.D.F. (2004). Covariate adjustment in randomized controlled trials with dichotomous outcomes increases statistical power and reduces sample size requirements. J. Clin. Epidemiol. 5, 454–460 10.1016/j.jclinepi.2003.09.014 15196615

[52] Xanthakis, V., Sullivan, L.M., Vasan, R.S., Benjamin, E.J., Massaro, J.M., D'Agostino Sr, R.B., and Pencina, M.J. (2014). Assessing the incremental predictive performance of novel biomarkers over standard predictors. Stat. Med. 15, 2577–2584 10.1002/sim.6165 PMC4047140 24719270

[53] Stiell, I.G., Wells, G.A., Vandemheen, K., Clement, C., Lesiuk, H., Laupacis, A., McKnight, R.D., Verbeek, R., Brison, R., and Cass, D. (2001). The Canadian CT Head Rule for patients with minor head injury. Lancet 9266, 1391–1396 10.1016/s0140-6736(00)04561-x 11356436

[54] Riley, R.D., Ensor, J., Snell, K.I.E., Debray, T.P.A., Altman, D.G., Moons, K.G.M., and Collins, G.S. (2016). External validation of clinical prediction models using big datasets from e-health records or IPD meta-analysis: opportunities and challenges. BMJ, 353, i3140 27334381 10.1136/bmj.i3140 PMC4916924

[55] Steyerberg, E.W., Nieboer, D., Debray, T.P.A., and van Houwelingen, H.C. (2019). Assessment of heterogeneity in an individual participant data meta-analysis of prediction models: An overview and illustration. Stat. Med. 22, 4290–4309 10.1002/sim.8296 PMC6772012 31373722

[56] Richter, S., Stevenson, S., Newman, T., Wilson, L., Menon, D.K., Maas, A.I., Nieboer, D., Lingsma, H.F., Steyerberg, E.W., and Newcombe, V.F. (2019). Handling of missing outcome data in traumatic brain injury research: a systematic review. J. Neurotrauma 36, 2743-2752 31062649 10.1089/neu.2018.6216 PMC6744946

[57] Van Buuren, S. (2018). Flexible imputation of Missing Data. CRC Press: New York, NY

[58] Royston, P., Altman, D.G., and Sauerbrei, W. (2006). Dichotomizing continuous predictors in multiple regression: a bad idea. Stat. Med. 25, 127–141 16217841 10.1002/sim.2331

