# Supplementary material for: Prognostic Research in Traumatic Brain Injury: Markers, Modeling, and Methodological Principles
Source: J Neurotrauma. 2021 Aug 23;38(18):2502–13. doi: 10.1089/neu.2019.6708 (PMC8403181; doi:10.1089/neu.2019.6708)
Supplement: Supplemental data [file Supp_Table1.pdf]

Supplementary Data

Supplementary Table S1. Prognostic Factors in the CRASH and IMPACT Prediction Models

Model	*CRASH*	*IMPACT*
Basic/Core	Age, GCS score, pupil reactivity, major extracranial injury	Age, motor score, pupil reactivity
CT model	Basic model plus: petechial hemorrhages, obliteration of the third ventricle or basal cisterns, subarachnoid hemorrhage, midline shift, unevacuated hematoma	Core model plus: hypoxia, hypotension, CT classification, presence of traumatic subarachnoid hemorrhage, epidural mass
Laboratory model	-	CT model plus: Glucose and hemoglobin concentrations

The outcome in the CRASH model was mortality at 14 days or unfavorable outcome at 6 months; the outcome for IMPACT was mortality or unfavorable outcome at 6 months.

CRASH, Corticosteroid Randomisation After Significant Head injury; IMPACT, International Mission for Prognosis and Clinical Trial design in Traumatic Brain Injury; GCS, Glasgow Coma Scale; CT, computed tomography.
