# Supplementary material for: Prognostic Research in Traumatic Brain Injury: Markers, Modeling, and Methodological Principles
Source: J Neurotrauma. 2021 Aug 23;38(18):2502–13. doi: 10.1089/neu.2019.6708 (PMC8403181; doi:10.1089/neu.2019.6708)
Supplement: Supplemental data [file Supp_Table4.pdf]

Supplementary Table S4. Examples of Known Prognostic Factors in TBI Studies

Categories	*Examples of prognostic factors*
Genetic constitution	Apolipoprotein E4 allele
Demographics	Age, sex
Clinical severity	Glasgow Coma Scale score, pupillary reactivity, extracranial injuries
Secondary insults	Hypotension (blood pressure), hypoxemia, hypothermia
Structural abnormalities	Marshall CT score and other CT classifications, type of lesions on CT or MRI (i.e. traumatic subarachnoid hemorrhage, epidural hematoma)
Laboratory parameters	Glucose, sodium, pH, coagulation parameters, hemoglobin
Blood biomarkers	NSE, S100B, GFAP, NF-L, t-Tau, UCHL1

Adapted from Winn, 2011[23]

CT, computed tomography; MRI, magnetic resonance imaging; NSE, neuron-specific enolase; S100B, S100 calcium-binding protein B; GFAP, glial fibrillary acidic protein; NF-L, neurofilament light; t-Tau, total Tau; UCHL1, ubiquitin carboxy-terminal hydrolase L1.
